# Ethanol Determination in *Post-Mortem* Samples: Correlation between Blood and Vitreous Humor Concentration

**DOI:** 10.3390/molecules25122724

**Published:** 2020-06-12

**Authors:** Fabio Savini, Angela Tartaglia, Ludovica Coccia, Danilo Palestini, Cristian D’Ovidio, Ugo de Grazia, Giuseppe Maria Merone, Elisa Bassotti, Marcello Locatelli

**Affiliations:** 1 Pharmatoxicology Laboratory—Hospital “Santo Spirito”, Via Fonte Romana 8, 65124 Pescara, Italy; fabio.savini@ausl.pe.it; 2 Department of Pharmacy, University of Chieti–Pescara “G. d’Annunzio”, Via dei Vestini 31, 66100 Chieti, Italy; angela.tartaglia@unich.it (A.T.); ludovica.coccia@studenti.unich.it (L.C.); 3 Head of Anti-Degradation Intervention Group (G.I.O.N.A.) of the Municipal Police of the City of Pescara, Commander f.f. of the Municipal Police of Pescara, Via del Circuito 26, 65100 Pescara, Italy; danilopalestini@gmail.com; 4 Department of Medicine and Aging Sciences, Section of Legal Medicine, University of Chieti–Pescara “G. d’Annunzio”, Via dei Vestini 31, 66100 Chieti, Italy; cridov@yahoo.it; 5 Fondazione IRCCS Istituto Neurologico Carlo Besta, Laboratory of Neurological Biochemistry and Neuropharmacology, Via Celoria 11, 20133 Milan, Italy; Ugo.DeGrazia@istituto-besta.it; 6 Department of Neuroscience, Imaging and Clinical Sciences, University of Chieti–Pescara “G. d’Annunzio”, Via dei Vestini 31, 66100 Chieti, Italy; giuseppe.merone@unich.it; 7 R&D Department Eureka Lab Division, 60033 Chiaravalle, Italy; bassotti@eurekaone.com

**Keywords:** ethanol, *post-mortem* analysis, blood, vitreous humor, specimens correlation, GC-FID

## Abstract

Ethanol (ethylic alcohol) represents the most commonly used drug worldwide and is often involved in clinical and forensic toxicology. Based on several reports, excessive alcohol consumption is the main contributing factor in traffic accidents, drownings, suicides, and other crimes. For these reasons, it becomes essential to analyze the alcohol concentration during autopsy. Although blood is usually used for alcohol analysis in *post-mortem* cases, it could suffer alterations, putrefaction, and microbial contaminations. As an alternative to whole blood, vitreous humor has been successfully used in medico-legal studies. In this work, *post-mortem* specimens were analyzed for ethanol determination. The analysis of blood and vitreous humor were carried-out using gas chromatography-flame ionized detector (GC-FID) with a total run time of 6 min. The method was validated in terms of limit of detection, limit of quantification, dynamic range, sensibility, recovery, precision and trueness. A linear regression analysis indicated a coefficient of determination (R^2^) of 0.9981. The study confirmed no statistically differences between alcohol concentration in blood and vitreous humor, leading vitreous humor as an excellent matrix that could be used as an alternative to whole blood in toxicological analysis in cases where blood is not available.

## 1. Introduction

Ethanol (ethylic alcohol) represents the most commonly used drug worldwide and often involved in clinical and forensic toxicology [1]. Ethanol (chemical formula C_2_H_6_O) is a psychoactive molecule that suppresses the activity of the central nervous system (CNS) by increasing the effects of γ-aminobutyric acid, or GABA [2]. According to a report released by the World Health Organization (WHO) in 2018, more than 3 million people died as a result of the harmful use of alcohol in 2016. Several studies report that excessive alcohol consumption is the main contributing factor in causing traffic accidents, drownings, suicides and other crimes. For these reasons, it becomes essential to analyze the alcohol concentration in post-mortem specimens. Globally an estimated 237 million men and 46 million women suffer from alcohol-use disorders with the highest prevalence among men and women in Europe (14.8% and 3.5%) and America (11.5% and 5.1%) [3]. In particular, in Italy, about 13,500 people aged 11 and over have the habit of consuming alcoholic beverages [4].

Being a small molecule, ethanol is quickly absorbed in all body tissues and fluids by simple diffusion; due to its water-soluble nature, the biological compartments with a high content of water, such as blood, urine, and vitreous humor present higher concentrations of ethanol. After ingestion of a single dose of ethanol, the time required to complete the absorption process ranges from 2 to 6 h, depending on different factors, such as ethanol amount ingested, the presence of food or other liquids, the functionality of the liver and biological variability [5]. Around 2–8% of ingested ethanol is eliminated through urine, sweat, or the breath; the other 92–98% is metabolized in the liver, by different enzyme systems. Ethanol is first transformed into acetaldehyde, which is then converted into acetic acid by acetaldehyde dehydrogenase.

**Figure 1:**
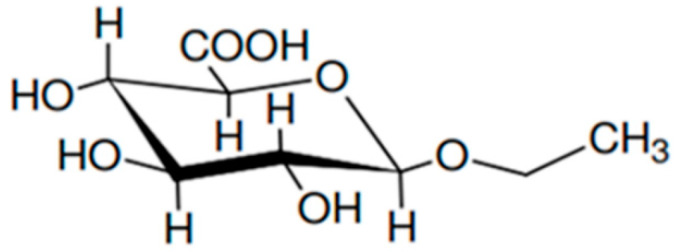
EtG chemical structure.

Since ethanol is rapidly eliminated, it is not a suitable compound for the analysis, and the research has focused on other alcohol markers, particularly ethyl glucuronide (EtG) and carbohydrate deficient transferrin (CDT). EtG (Figure 1) is a direct phase II metabolite of ethanol formed after conjugation with glucuronic acid via UDP-glucuronosyltransferases [6]. Ethyl glucuronide (EtG), being a direct metabolite of ethanol, it may be used to differentiate between ante-mortem alcohol intake and post-mortem formation due to putrefactive processes. In fact, in some cases, alcohol might be produced after death by microbial activity or fermentation of glucose [4,7]. Carbohydrate deficient transferrin (CDT) is an indirect marker used for identifying recent and regular consumption of ethanol. The transferrin glycoform with the highest diagnostic sensitivity is disialotrasferrin [8].

The most used specimen for alcohol *post-mortem* analysis is whole blood [4]. Usually chemical preservative is added to inhibit post-mortem alcohol formation. Preservation of blood samples with sodium or potassium fluoride (1–2% *w/v*) is routine in most laboratories. The fluoride ions are enzyme inhibitors that avoid ethanol production between the time of the autopsy and sample analysis in the lab [7]. However, whole blood could be subject to different post-mortem changes. First, in *post-mortem* blood samples the water content changes from 60% to 90% and pH value differ significantly from physiological ranges [9]. Physiologically blood pH is regulated by acid-base buffer and ranges from 7.35 to 7.45. After death, the buffering system is not maintained, and blood pH changes can occur different studies showed that after death blood pH change from 7.0 to 5.5 [10]. Furthermore, depending on the time between the death and the collection of the sample, the consistency of the blood changes with coagulation [11]. Additionally, after death, the oxygen available in the body is depleted and the cells become anaerobic, losing energy to maintain their membrane gradients. This lack of selectivity in the membrane channels causes a release of substances previously contained within cell compartments in extra-cellular space with consequent hemolysis [12]. This loss of membranes cell integrity causes tissue liquefaction with consequent invasion by bacteria and an increase in glucose concentration, which represents the simplest substrate for ethanol synthesis. In addition, the detection of *post-mortem* ethanol is often confounded with *post-mortem* production of ethanol; in fact, different species of bacteria, yeast and molds are able of producing ethanol from many substrates. The possibility of *post-mortem* ethanol formation by these species increases with other factors, such as the storage temperature or the time between death and autopsy, and is not easy to distinguish between *post-mortem* ethanol production and *ante-mortem* alcohol assimilation.

To overcome this limitation, the use of an alternative/complementary specimen to the blood is of great relevance for forensic purposes. Vitreous humor (VH) is one of the biological specimens widely used in forensic toxicology, mostly when the body is severely damaged or affected by putrefaction [1]. Vitreous humor is a gelatinous substance that consists of 98% H_2_O and of 2% collagen fibers, glycosaminoglycans as hyaluronic acid, cells, electrolytes, carbohydrates and other proteins [13]. Vitreous humor is considered advantageous because due to eyeball is less exposed to bacterial contamination, it is easy to sample (VH is sampled by syringe from the center of the eyeball with slow aspiration) and shows sample stability over time after death [14,15]. Additionally, vitreous humor is less subject to *post-mortem* ethanol formation: being encapsulated in the eyeball the eye water is less subject to *post-mortem* alcohol generation and the ethanol levels in vitreous humor remain constant. However, some limitations are present also for VH such as a limited volume of samples and the presence of the retinal blood barrier, which limits the passage inside and outside.

In this work, following our previous work on whole blood and non-conventional matrices [16,17,18,19,20,21] was validated a new, fast, high-throughput, and reproducible GC-FID assay for the evaluation of the ethanol concentration in blood and vitreous humor. The purpose was to highlight that the new method is a valid and validated alternative to other procedures, confirming the correlation between these different specimens, as reported in literature [22,23,24,25], that allow estimating blood alcohol concentrations when blood is unavailable or contaminated or in cases where blood analysis cannot be carried out.

## 2. Results and Discussion

### 2.1. Method Validation

The reported method was validated in terms of the limit of detection, limit of quantification, dynamic range, sensibility, recovery, precision and trueness. The procedure satisfies the International Guidelines for Bioanalytical methods and the guidelines for laboratories for the analysis of substances of abuse for toxicological-forensic and medico-legal purposes (revision 3 of 1 March 2010 by the Quality Commission1 of the Italian Forensic Toxicologists Group (GTFI).

The dynamic range was found between 0.01 and 10 g/L with a regression coefficient of 0.9981. The limit of detection (LOD) was 0.003 g/L, whereas the limit of quantification (LOQ) was 0.01 g/L.

Briefly, precision at 0.5 g/L was 6.8%. Trueness was checked at three different concentrations, 0.01 g/L, 0.25 g/L and 2 g/L and deviations were within the 20% for the first value (LOQ) and within the 15% for the other 2 values. No interferences were found in all analyzed specimens. All specifications on the method validation can be found from Eureka Srl—Lab Division, code GC73010. Particularly, the intra- and inter-day precision and trueness were found as reported in Table 1, also with the figure of merits of the herein validated procedure.

Generally, an attempt is made to increase the concentrations included in the linearity interval until the mathematical model shows response linearity.

**Figure 2:**
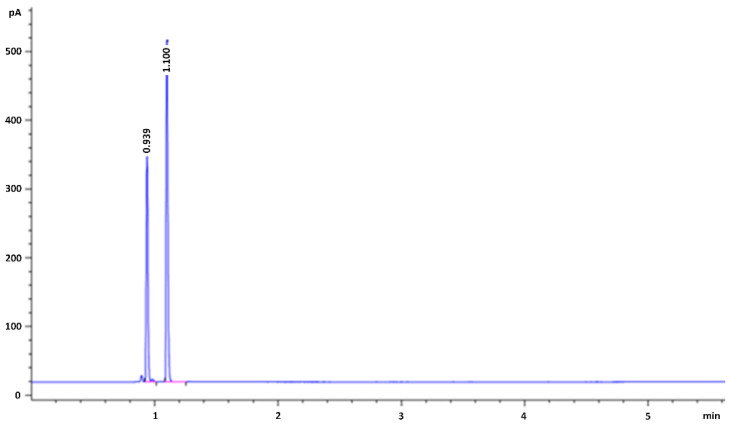
Representative chromatogram of blood sample using GC-FID with HP-Innowax column of ethanol (first peak, 0.939 min as retention time) and *n*-propanol (IS, second peak, 1.1 min as retention time).

In Figure 2 it is shown that the chromatogram obtained from analysis conducted in the gas chromatography-flame ionization detector (GC-FID) where ethanol concentration in blood was 1.31 g/L (sample 1). The analytes were identified by comparison of the retention times with those of standard solutions. As indicated in the chromatogram, ethanol retention time was 0.939 min, while *n*-propanol (IS) retention time was 1.1 min. The total run time was 6 min. No carry over phenomena was observed during the calibration, quality control samples (QCs) and real samples analysis.

To prevent further *post-mortem* ethanol production, a chemical preservative, sodium fluoride, was added. The fluoride ion is effective in inhibiting the activity of different types of enzymes including enolase, a component of the glycolytic pathway, and this is important for the action of the yeasts, fungi and microorganisms responsible for fermentation.

### 2.2. Ethanol Concentration in Blood and Vitreous Humor

The measurement of the blood alcohol concentration is a routine procedure during the autopsy, important in the legal procedure to determine the state of sobriety of the deceased subject. Given the importance of this measurement, it is essential to have additional specimens to analyze in order to obtain this information, for example vitreous humor (VH). In Table 2 is reported the vitreous humor alcohol concentration (VAC) and blood alcohol concentration (BAC) expressed in g/L of 31 *post-mortem* specimens analyzed in this study, and the corresponding standard deviations (*n* = 3). *Post-mortem* specimens’ examinations were carried out at the Pharmatoxicology Laboratory—Hospital “Santo Spirito”, Pescara (Italy). The corresponding ratio between vitreous humor and blood concentration was also calculated and reported (Table 2). The VAC ranged between 0.63 and 2.78 g/L, while BAC ranged between 0.35 and 2.6 g/L.

The results show that in 23 of 31 analyzed specimens (74%) ethanol concentration in vitreous humor was slightly higher than in blood, according to the previous study [23] The BAC exceeded VAC in eight cases (26%).

Due to its solubility and low molecular weight, ethanol is rapidly absorbed and distributed in all body tissue and fluids according to the water content of a different compartment; the vitreous humor has a water content of 98.7% against 80.0% of water content in blood: for this reason VAC are usually 10–20% higher than BAC. However, different reasons could influence the BAC, mostly post-mortem production of alcohol must be considered. This *post-mortem* production depends on different factors such as the temperature, the post-mortem interval, the species of microorganism and the trauma to the body. Alcohol concentration in vitreous humor is not influenced by the formation of alcohol during the putrefaction process and remains stable after death for a longer period of time.

**Figure 3:**
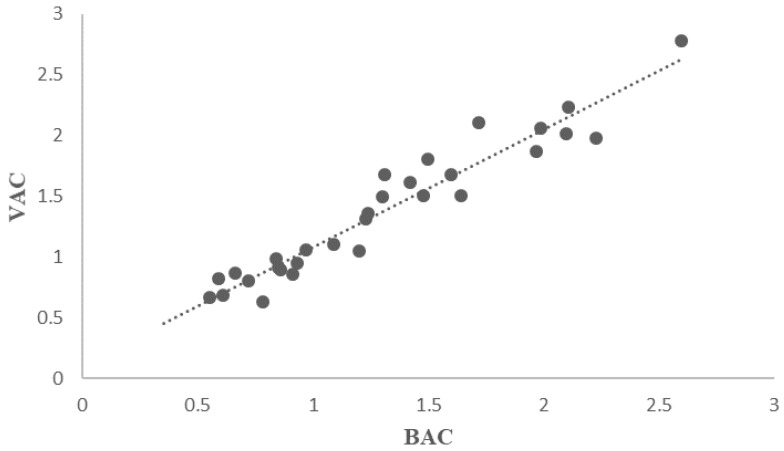
Correlation BAC (g/L)–VAC (g/L).

In order to establish the relationship between alcohol concentration in both examined fluids, statistical analysis was performed, and a correlation coefficient and regression equation were calculated. Data analysis was performed by linear regression (y=mx+b) and Figure 3 shows the linear correlation between the ethanol concentrations in vitreous humor *versus* ethanol concentrations in blood. The regression analysis yielded the equation

y=1.01 x+0.0479 g/L

where y= vitreous humor alcohol concentration and x= blood alcohol concentration, respectively. In particular, the value m=1.01 (b=0.0479 g/L represents a very small value) indicates the presence of direct proportionality between the concentration in two different specimens analyzed in the study.

In this study, the coefficient of determination (R^2^) was calculated at 0.9227, which shows that the linear model can be applied to correlate the BAC and VAC concentrations.

Statistical analysis shows a strong correlation between the concentration of ethanol in blood and vitreous humor. The present study confirms the opinion concerning the very high degree of correlation between the concentration of alcohol in the blood and the vitreous humor.

## 3. Materials and Methods

### 3.1. Reagents and Solvents

The study was conducted at the Pharmacology Laboratory, Hospital “Santo Spirito” of Pescara, Italy during 2019 on 31 forensic cases in which samples of blood and vitreous humor were collected for toxicological examination. Ethanol (20.0 g/L), *n*-propanol, used as Internal Standard (IS) and lyophilized whole blood was purchased from Eureka Lab Division (purities ≥ 99.9%). The lyophilized whole blood is easy to use after reconstituting with water.

### 3.2. Samples Collection and Preparation

Toxicological tests were carried out on 31 *post-mortem* whole blood and vitreous humor samples from suspected ethanol poisoning. Blood samples were collected before opening the body, mainly from the femoral vein to avoid contamination of the blood with other fluid. Vitreous humor was collected from both eyes by puncturing the eyeballs with a thin needle. All specimens were stored at 4 °C with the addition of sodium fluoride (in the ratio of 100 mg/10 mL of sample volume) and analysis were carried out within 5 days after collection. Samples were placed in headspace vials, hermetically closed, and ethanol in various concentrations was added using Hamilton syringe 50 µL. The general procedure can be set as follow: 50 µL reconstituted matrix + 50 µL ethanol 0.5 g/L; 50 µL reconstituted matrix + 50 µL ethanol 1.0 g/L and 50 µL reconstituted matrix + 50 µL ethanol 2.0 g/L. Vials were a blank sample that was prepared with 50 µL of reconstituted blood/vitreous humor + 50 µL of HPLC H_2_O. Of diluted internal standard (*n*-propanol) 100 µL was added to all vials with a Hamilton syringe of 100 µL. Samples were vortex for 10 min and were injected by a headspace autosampler into the GC-FID instrument.

### 3.3. Chromatographic Analysis

Quantitative determination of ethanol was carried out using GC-FID. An Agilent 7820 A series GC instrument and HP-Innowax (30 m × 0.25 mm × 0.25 μm) capillary column were used. Helium (flow = 1.5 mL/min) was used as carrier gas. The injector, the column and the detector were maintained at 250 °C, 40 °C and 250 °C, respectively. The analysis was carried out by isothermal elution. *n*-Propanol was used as internal standard. Nitrogen, hydrogen and air were used as gasses for the FID detector at 25, 40 and 400 mL/min, respectively. The autosampler parameters have been set as follows: syringe temperature of 60 °C and vortex temperature of 50 °C. Headspace analysis was selected, and the sample was maintained under stirring for 30 sec during the analyte extraction.

### 3.4. Method Validation

The validation of the analytical method was carried out according to the International Guidelines [26,27,28] in order to check linearity, LOD, LOQ, selectivity and recovery.

## 4. Conclusions

This study confirms that there is a correlation between the concentration of ethanol in vitreous humor and the concentration of ethanol in blood, as measured by the linear regression analysis. For this reason, when blood is unavailable or contaminated, the vitreous humor represents an excellent matrix that could be used as an alternative. Moreover, vitreous humor presents different advantages because it is a matrix scarcely contaminated by microorganisms, less prone to decomposition and distribution *post-mortem*, it is easy to collect, needs easier pre-treatment, has relatively few compounds interfering with the analytical process and shows the stability of the compound over time after death. The herein proposed method allows directing quantifying the ethanol in both specimens without carry over phenomena, and particularly, under isothermal conditions, removing the transferability method drawbacks. Furthermore, this procedure fulfills with the actual guidelines, respecting the limits requested for an analytical procedure applied in forensic analysis.

## Figures and Tables

**Table 1:** Method validation parameters.

	**INTRA-DAY**	
	**Precision (RSD%)**	**Trueness (Bias%)**
0.01 g/L	7.9	9.2
0.5 g/L	6.8	7.1
2 g/L	5.2	5.9
	**INTER-DAY**	
0.01 g/L	8.9	10.5
0.5 g/L	7.6	8.1
2 g/L	5.6	6.1
**LLOD**	0.003 g/L	
**LLOQ**	0.01 g/L	
**Linearity**	0.01–10 g/L	
**r^2^**	0.9981 ± 0.0025	

**Table 2:** Vitreous humor alcohol concentration (VAC) and blood alcohol concentration (BAC) for 31 post-mortem specimens. Data are reported as mean ± standard deviation (*n* = 3).

Samples	VAC (g/L)	BAC (g/L)	VAC/BAC RATIO
1	1.68 ± 0.09	1.31 ± 0.06	1.28 ± 0.09
2	< LOQ	0.35 ± 0.03	-
3	0.91 ± 0.04	0.85 ± 0.04	1.07 ± 0.07
4	2.23 ± 0.19	2.11 ± 0.11	1.06 ± 0.11
5	1.5 ± 0.09	1.64 ± 0.12	1.91 ± 0.18
6	0.95 ± 0.06	0.93 ± 0.04	1.02 ± 0.08
7	1.61 ± 0.08	1.42 ± 0.07	1.13 ± 0.08
8	1.31 ± 0.09	1.23 ± 0.06	1.07 ± 0.09
9	1.5 ± 0.11	1.48 ± 0.09	1.01 ± 0.10
10	2.01 ± 0.18	2.10 ± 0.16	0.96 ± 0.11
11	1.87 ± 0.12	1.97 ± 0.14	0.95 ± 0.09
12	1.36 ± 0.09	1.24 ± 0.05	1.10 ± 0.09
13	0.86 ± 0.04	0.91 ± 0.03	0.95 ± 0.05
14	1.98 ± 0.16	2.23 ± 0.15	0.89 ± 0.09
15	0.82 ± 0.06	0.59 ± 0.04	1.39 ± 0.14
16	1.49 ± 0.07	1.30 ± 0.08	1.15 ± 0.09
17	2.78 ±0.17	2.60 ± 0.17	1.07 ± 0.10
18	1.05 ± 0.08	1.20 ± 0.08	0.88 ± 0.09
19	1.68 ± 0.11	1.60 ± 0.11	1.05 ± 0.10
20	0.98 ± 0.04	0.84 ± 0.03	1.17 ± 0.06
21	0.67 ± 0.03	0.55 ± 0.02	1.22 ± 0.07
22	1.10 ± 0.05	1.09 ± 0.05	1.01 ± 0.07
23	2.06 ± 0.13	1.99 ± 0.13	1.04 ± 0.09
24	0.80 ± 0.07	0.72 ± 0.03	1.11 ± 0.11
25	1.06 ± 0.05	0.97 ± 0.09	1.09 ± 0.11
26	0.87 ± 0.04	0.66 ± 0.06	1.32 ± 0.13
27	2.10 ± 0.16	1.72 ± 0.09	1.22 ± 0.11
28	1.80 ± 0.11	1.50 ± 0.07	1.20 ± 0.09
29	0.68 ± 0.05	0.61 ± 0.02	1.11 ± 0.09
30	0.89 ± 0.06	0.86 ± 0.04	1.03 ± 0.08
31	0.63 ± 0.06	0.78 ± 0.03	0.81 ± 0.08

<LOQ: below the limit of quantification.

## Abbreviations

All the abbreviations are given in the text, for extended, the first time they are used.
